# Supplementary material for: Mechanism of regulation of the Helicobacter pylori Cagβ ATPase by CagZ
Source: Nat Commun. 2023 Jan 30;14:479. doi: 10.1038/s41467-023-36218-4 (PMC9886983; doi:10.1038/s41467-023-36218-4)
Supplement: Supplementary file 1 — Supplementary information [file 41467_2023_36218_MOESM1_ESM.pdf]

Supplementary Information

Mechanism of regulation of the *Helicobacter pylori* Cagβ ATPase by CagZ

Wu et al

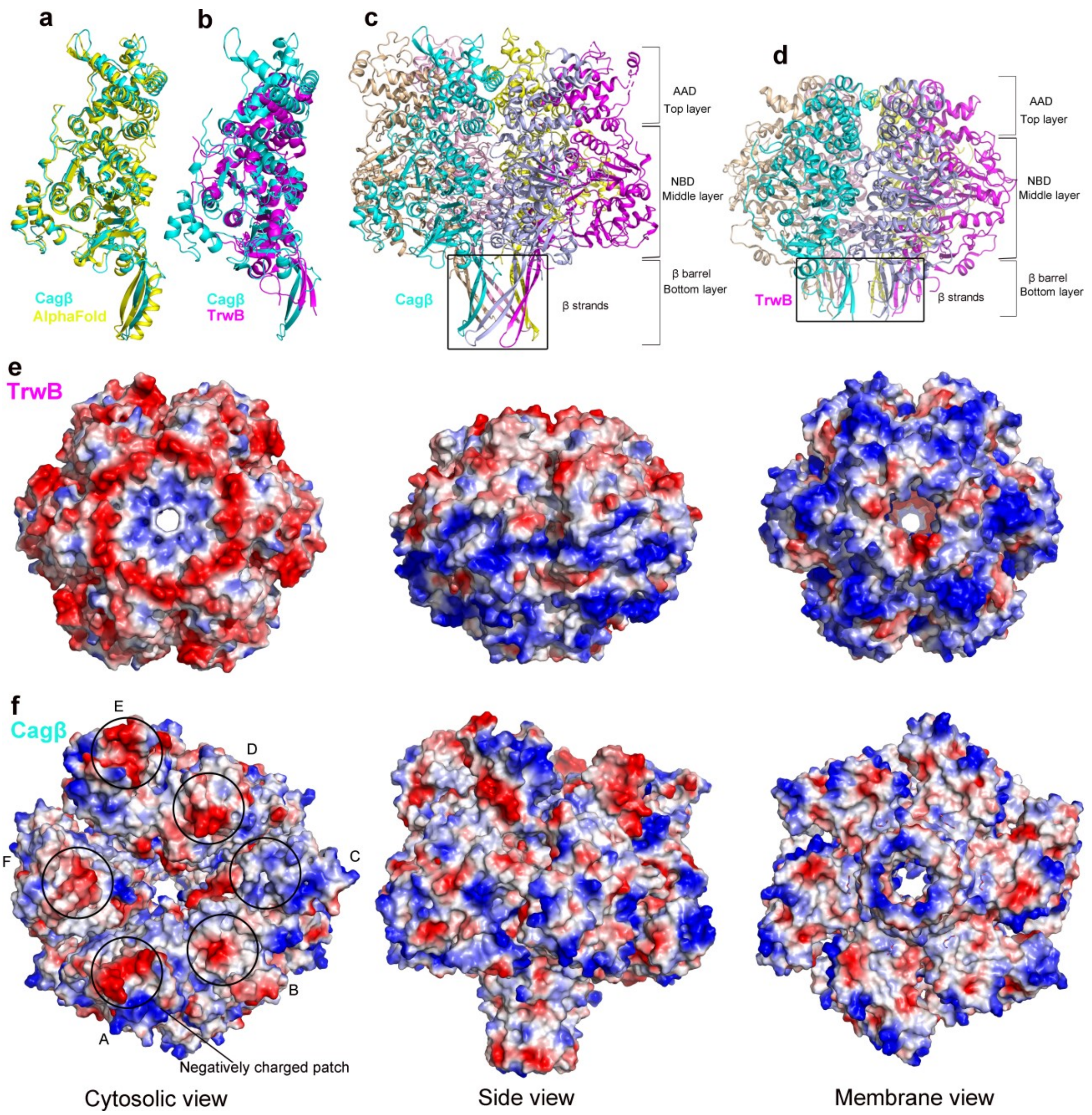

Supplementary Figure 1. Structural comparison of Cagβ with TrwB and the AlphaFold model. a, Structural comparison of the Cagβ protomer with the AlphaFold model (AF-O25260-F1) **b,** Structural comparison of the Cagβ protomer with TrwB (PDB ID: 1e9r). **c, d,** Ribbon presentation of Cagβ and TrwB hexamer with the three layers labeled**. e,** Surface electrostatic potential of the TrwB hexamer in the cytosolic view (left panel), side view (middle panel) and membrane view (right panel). **f,** Surface electrostatic potential of the Cagβ hexamer in the same views as in e. In the cytosolic view, the negatively charged patches on AAD are highlighted with circles. The negatively charged patch is obvious in protomers A, B D, E and F. This patch appears to be absent in protomer C, because the corresponding region in protomer C is disordered and not included in the atomic model.

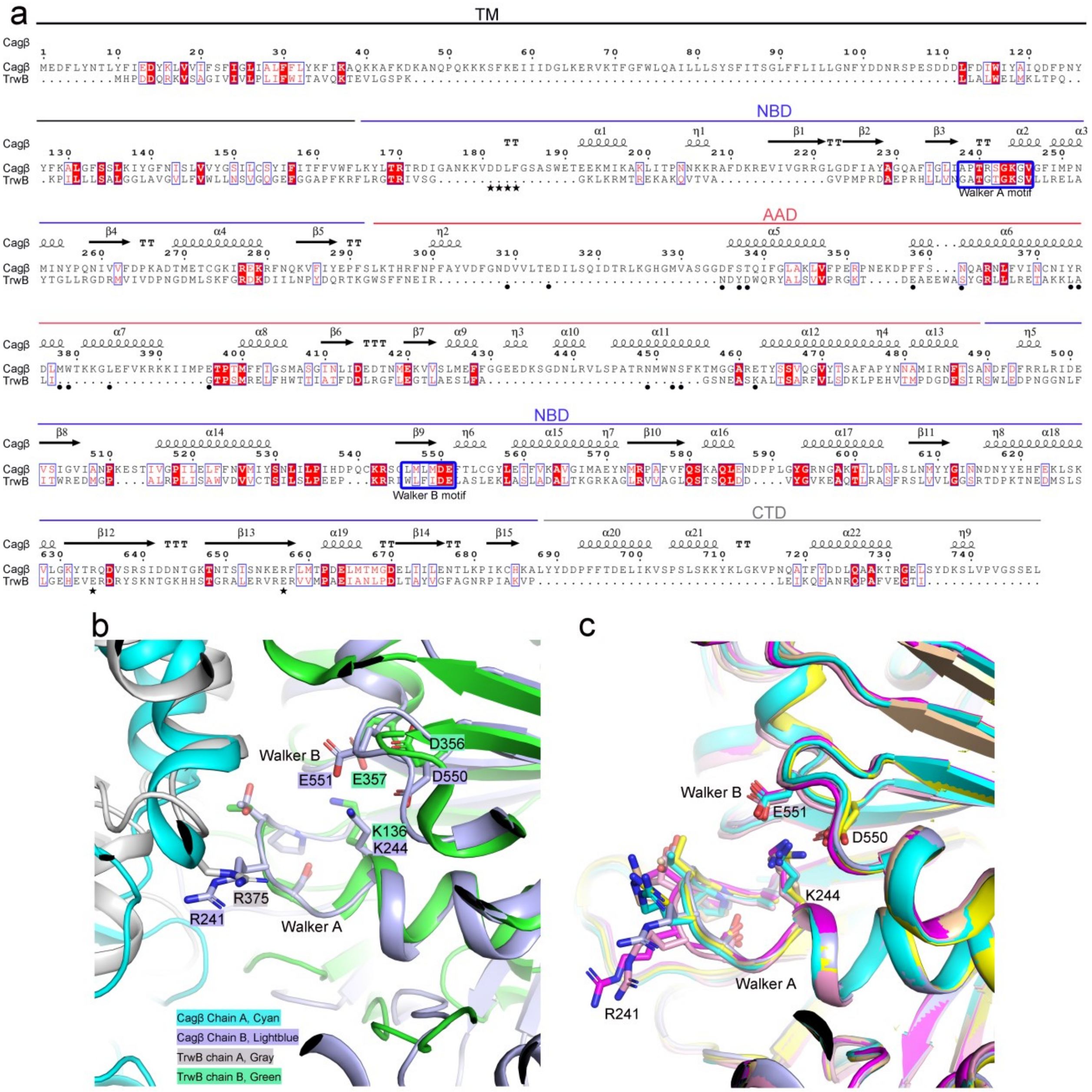

Supplementary Figure 2. Structure-based sequence alignment of Cagβ and TrwB. a, Cagβ shows a high degree of sequence divergence from TrwB, particularly in the AAD, CTD and TM. However, the NBD region is much more conserved between two proteins. Red boxes indicate the Walker A and B motifs. Stars highlight D181, D182, L183, F184 and R634, R657 that mediate inter-subunit interactions at the β-barrel. Residues involved in interactions with CagZ are highlighted with black circles. **b,** Structure overlay of the ATP binding site between Cagβ and TrwB. **c**, Structure overlay of the ATP binding site of the six Cagβ protomers.

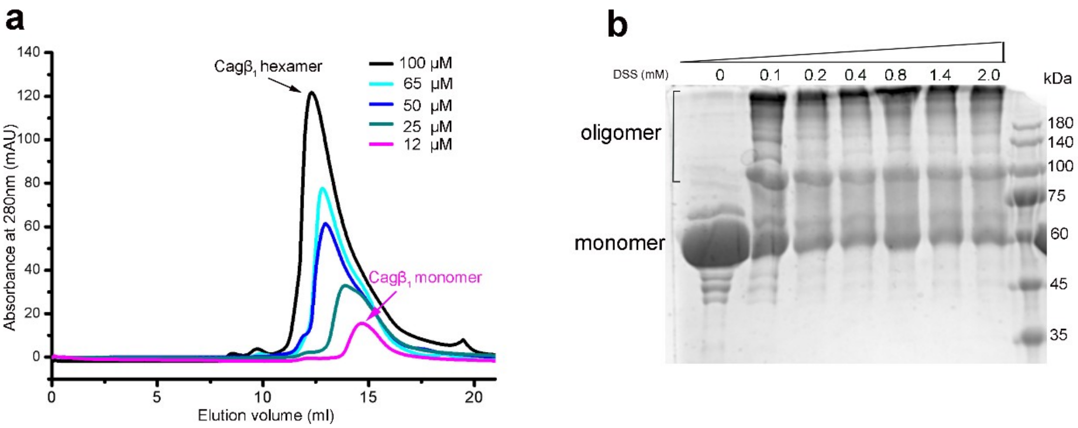

Supplementary Figure 3. Cagβ oligomerizes in vitro. a, Size exclusion chromatography analysis of purified Cagβ_1 at different concentrations. The data shows that Cagβ_1 undergoes concentration-dependent oligomerization. **b,** Cross-linking assays of Cagβ_1 oligomerization. The protein was cross-linked with various concentrations of DSS and analyzed with SDS-PAGE.

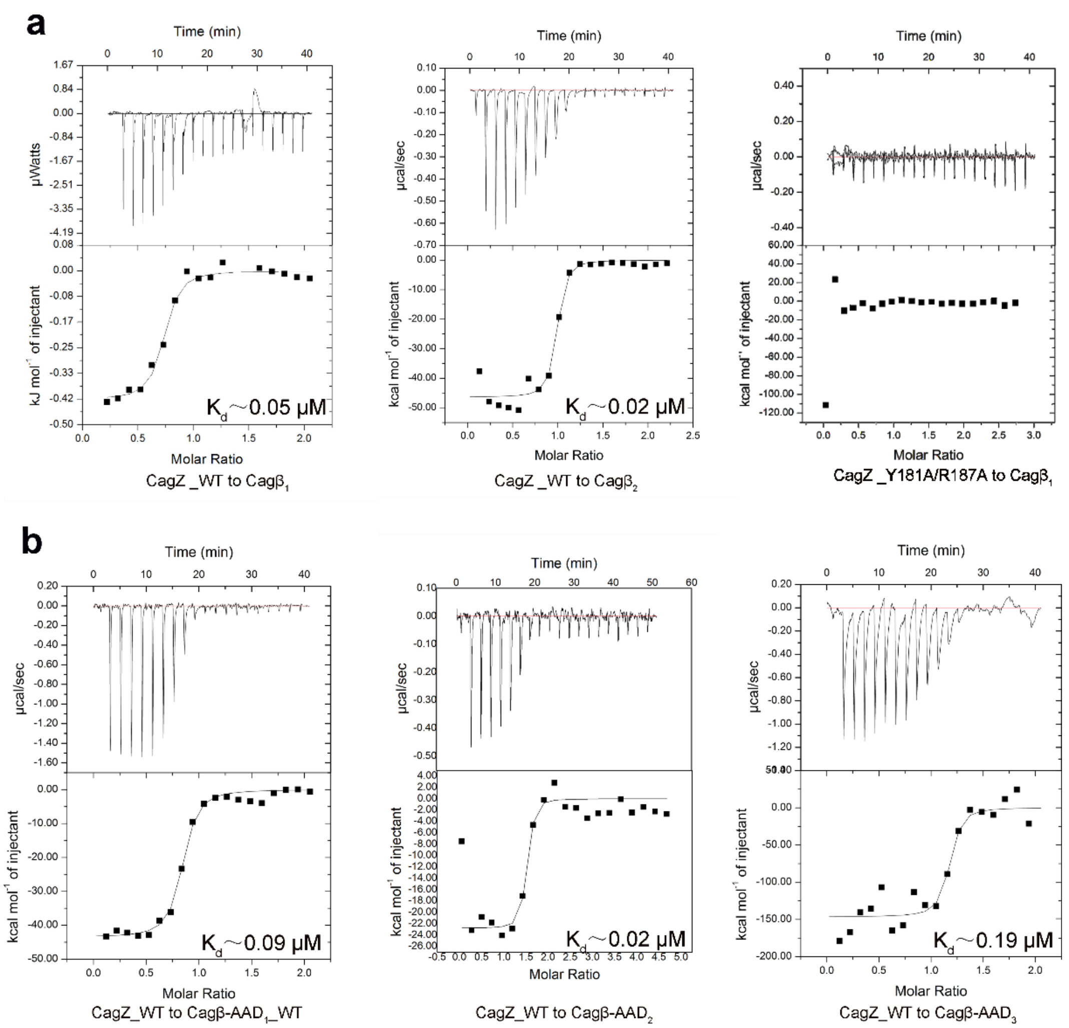

Supplementary Figure 4. ITC binding assays of the interaction between CagZ and Cagβ.
a, The K_d between CagZ-WT or it's mutant and Cagβ-WT. **b**, The K_d between CagZ-WT and Cagβ AAD truncations.

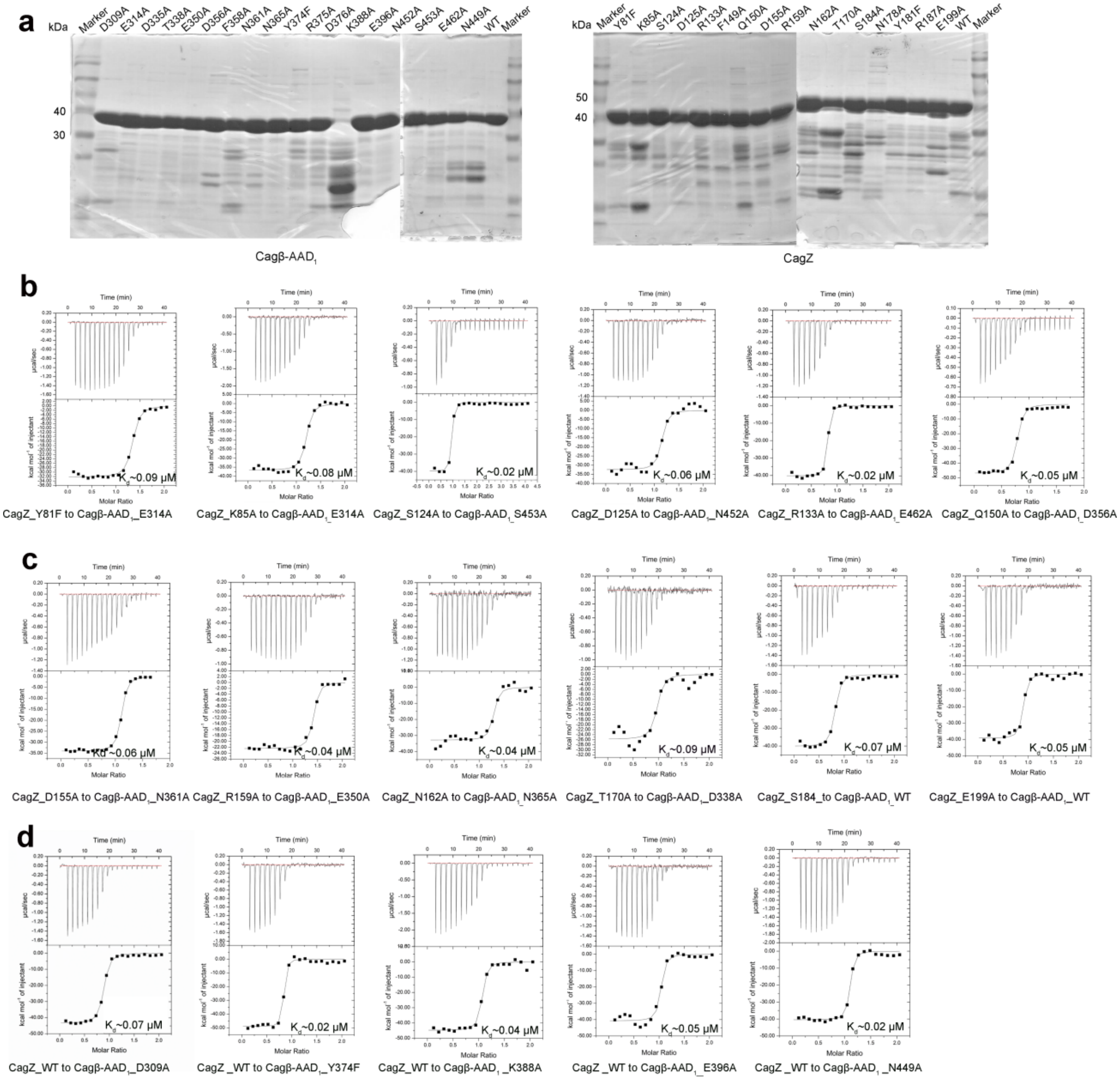

Supplementary Figure 5. Additional mutational analyses of the binding interface between CagZ and Cagβ-AAD_1. **a**, SDS-PAGE analyses of purified protein used in the ITC experiments. **b-d**, ITC assays of the interaction between variants of CagZ and Cagβ-AAD_1. The results show that most of mutations at the base or the right side of the U-shaped binding interface have no or weak effects on the binding between CagZ and Cagβ-AAD_1.

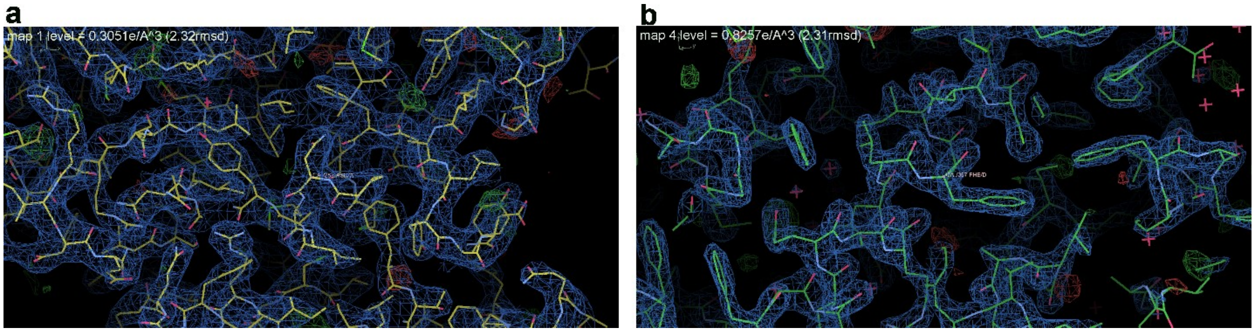

Supplementary Figure 6. Representative density map for Cagβ$_1$ hexamer (a) and Cagβ$_{1}$-AAD/CagZ (b).

Supplementary Table 1. Data Collection, Phasing, and Refinement Statistics

Date Collection	Cagβ$_1$	Cagβ-AAD$_1$/CagZ complex
Space group	P12$_1$1	P4$_1$2$_1$2
Cell parameters		
a, b, c (Å)	112.11,163.28,112.22	96.30, 96.30, 212.66
α,β,γ(°)	90.00,93.33,90.00	90.00, 90.00, 90.00
Resolution (Å)	50.00-2.8	49.11-2.00
R merge (%)	0.079(1.026)	0.083(0.835)
I/σI	1.88	1.15
Completeness	99.23(99.30)	99.9 (99.9)
Wilson B-factor (Å^2)	77.4	34.4
Refinement		
Resolution (Å)	40.79-2.8(2.87-2.8)	49.11-2.10 (2.15-2.10)
No. reflections	97,785(1,988)	64,937 (2,346)
R work /R free (%)	19.70(24.67)	19.20/23.20
No. atoms		
Protein	24484	6544
Water	11	522
R.m.s.d. bonds (Å)	0.003	0.007
R.m.s.d. angles (°)	0.555	0.803
Ramachandran plot		
Favored (%)	95.42	97.96
Allowed (%)	4.39	1.90
Outliers (%)	0.19	0.14
Rotamer outliers (%)	2.64	0.00

Values in parentheses refer to the highest resolution shell. R.m.s.d., root mean square deviation.

Supplementary Table 2. Expression, purification of Cagβ constructs and binding with CagZ.

Cagβ constructs	expression[a]	purification[b]	cagZ binding[c] (μM)	1σ (μM)
lacking the transmembrane domain truncations				
$Cag\beta_1$(166-748)	++	++	0.05	0.04-0.08
$Cag\beta_2$ (243-748)	+	+	0.02	0.01-0.05
$Cag\beta_3$ (260-748)	+	-	n.a.	
$Cag\beta_4$ (335-748)	+	-	n.a.	
$Cag\beta_5$ (380-748)	++	-	n.a.	
$Cag\beta_6$ (460-748)	++	-	n.a.	
$Cag\beta_7$ (540-748)	+	++	-	
$Cag\beta_8$ (591-748)	+++	+++	-	
$Cag\beta_9$ (630-748)	+++	+++	-	
$Cag\beta_{10}$ (701-748)	+++	+++	-	
AAD domain truncations				
Cagβ-AAD_1 (289-488)	+++	+++	0.09	0.08-0.11
Cagβ-AAD_2 (289-505)	+++	+++	0.02	0.01-0.34
Cagβ-AAD_3 (299-488)	+++	+++	0.19	0.09--4.55

The expression levels of all the constructs were assessed by SDS-PAGE analyses of whole cell lysates. The levels of purified proteins were analyzed after Ni-NTA purification. n.a. indicates no attempt.

Supplementary Table 3. Primers used in this study

Primers	Sequence
Cagβ(166) -F-pet32a	CGGGATCCAAATACTTAACTCGGACTAGAGATATAGGAG
Cagβ(243) -F-pet32a	CGGGATCCGGTAAGGGGGTGGGTTTCATCATG
Cagβ(260) -F-pet32a	CGGGATCCATCGTTGTGTTTGACCCTAAAG
Cagβ(289) -F-pet32a	CGGGATCCGAACCTTTCTCCTTAAAAACACAC
Cagβ(299) -F-pet32a	CGGGATCCAATCCTTTCGCTTATGTGGATTTTGGTAATG
Cagβ(335) -F-pet32a	CGGGATCCGATTTTTCCACTCAAATCTTTG
Cagβ(380) -F-pet32a	CGGGATCCACTAAAAAGGGGCTTGAGTTTG
Cagβ(460) -F-pet32a	CGGGATCCGCTAGAGAAACTTATAGCTC
Cagβ(540) -F-pet32a	CGGGATCCCAGTGCAAAAGAAGTTGCTTGATG
Cagβ(591) -F-pet32a	CGGGATCCCTTGGTTATGGTAGGAATG
Cagβ(630) -F-pet32a	CCCGGATCCGGGAAATACACAAGGCAAGACGTGAG
Cagβ(488) -R-pet32a	CCGCTCGAGTTAGCTCGTGAAATTTCTAATCATTG
Cagβ(505) -R-pet32a	CCGCTCGAGTTAACCAATACTCACTTCATCGATCCTTAAACG
Cagβ(748) -R-pet32a	CCCTCGAGTCACAGTTCACTTGAACCCACAGGC
CagZ(2) -F-pet32a/pet28a	CGGGATCCGAACTCGGTTTCAATGAAGCAGAAAG
CagZ(199) -R-pet32a/pet28a	CCGCTCGAGTTATTCCAAATTTAATTTTAATTGGGTTATC
CagZ(Y81F)-F-pet32a	CTTGTGTTGGCCAGTTTCAATCGTTGCAAACAAG
CagZ(Y81F)-R-pet32a	CTTGTTTGCAACGATTGAAACTGGCCAACACAAG
CagZ(K85A)-F-pet32a	CAGTTACAATCGTTGCGCACAAGAAAAGACTTTTGC
CagZ(K85A)-R-pet32a	GCAAAAGTCTTTTCTTGTGCGCAACGATTGTAACTG
CagZ(S124A)-F-pet32a	GAAGTGGCAAGAATTGCAGACCTAGTGGCTTCTTATC
CagZ(S124A)-R-pet32a	GATAAGAAGCCACTAGGTCTGCAATTCTTGCCACTTC
CagZ(D125A)-F-pet32a	GTGGCAAGAATTTCAGCCCTAGTGGCTTCTTATCTG
CagZ(D125A)-R-pet32a	CAGATAAGAAGCCACTAGGGCTGAAATTCTTGCCAC
CagZ(R133A)-F-pet32a	GCTTCTTATCTGCCAGCAGAGTATTTACCGCCATTC
CagZ(R133A)-R-pet32a	GAATGGCGGTAAATACTCTGCTGGCAGATAAGAAGC
CagZ(F149A)-F-pet32a	GCAATATGATGGGCGTGGCGGCTCAGATCCTAGGGATAG
CagZ(F149A)-R-pet32a	CTATCCCTAGGATCTGAGCCGCCACGCCCATCATATTGC
CagZ(Q150A)-F-pet32a	GATGGGCGTGGCGTTTGCGATCCTAGGGATAGATG
CagZ(Q150A)-R-pet32a	CATCTATCCCTAGGATCGCAAACGCCACGCCCATC
CagZ(D155A)-F-pet32a	CAGATCCTAGGGATAGCTGATTTTGGAAGGAAGC
CagZ(D155A)-R-pet32a	GCTTCCTTCCAAAATCAGCTATCCCTAGGATCTG
CagZ(R159A)-F-pet32a	GATAGATGATTTTGGAGCGAAGCTCAATGAGATTG
CagZ(R159A)-R-pet32a	CAATCTCATTGAGCTTCGCTCCAAAATCATCTATC
CagZ(N162A)-F-pet32a	GATTTTGGAAGGAAGCTCGCTGAGATTGTCCAAGATATAG
CagZ(N162A)-R-pet32a	CTATATCTTGGACAATCTCAGCGAGCTTCCTTCCAAAATC

CagZ(Q166A)-F-pet32a	GCTCAATGAGATTGTCGCAGATATAGGGACTA
CagZ(Q166A)-R-pet32a	TAGTCCCTATATCTGCGACAATCTCATTGAGC
CagZ(T170A)-F-pet32a	GTCCAAGATATAGGGGCTAAATATATTATTTTGAG
CagZ(T170A)-R-pet32a	CTCAAAATAATATATTTAGCCCCTATATCTTGGAC
CagZ(N178A)-F-pet32a	TATTATTTTGAGCAAAGCTAAGACTTATCTCACTTC
CagZ(N178A)-R-pet32a	GAAGTGAGATAAGTCTTAGCTTTGCTCAAAATAATA
CagZ(Y181A)-F-pet32a	GAGCAAAAATAAGACTGCTCTCACTTCTTTAGAAAG
CagZ(Y181A)-R-pet32a	CTTTCTAAAGAAGTGAGAGCAGTCTTATTTTTGCTC
CagZ(S184A)-F-pet32a	GACTTATCTCACTGCTTTAGAAAGAGCTAAATTG
CagZ(S184A)-R-pet32a	CAATTTAGCTCTTTCTAAAGCAGTGAGATAAGTC
CagZ(R187A)-F-pet32a	CTCACTTCTTTAGAAGCAGCTAAATTGATAACC
CagZ(R187A)-R-pet32a	GGTTATCAATTTAGCTGCTTCTAAAGAAGTGAG
CagZ(E199A)-F-pet32a	CCAATTAAAATTAAATTTGGCATAACTCGAGCACCAC
CagZ(E199A)-R-pet32a	GTGGTGCTCGAGTTATGCCAAATTTAATTTTAATTGG
CagZ N178A(Y181A)-F-pet32a	TATTATTTGAGCAAAGCTAAGACTGCTCTCACTTC
CagZ N178A(Y181A)-R-pet32a	GAAGTGAGAGCAGTCTTAGCTTTGCTCAAATAATA
Cagβ(D309A)-F-pet32a	GTGGATTTTGGTAATGCTGTGGTTTTGACCGAAG
Cagβ(D309A)-R-pet32a	CTTCGGTCAAAACCACAGCATTACCAAAATCCAC
Cagβ(E314A)-F-pet32a	GATGTGGTTTTGACCGCAGACATACTCTCTCAAATTG
Cagβ(E314A)-R-pet32a	CAATTTGAGAGAGTATGTCTGCGGTCAAAACCACATC
Cagβ(D335A)-F-pet32a	GTGGCTAGTGGAGGGGCTTTTTCCACTCAAATC
Cagβ(D335A)-R-pet32a	GATTTGAGTGGAAAAAGCCCCTCCACTAGCCAC
Cagβ(S337A)-F-pet32a	GTGGAGGGGATTTTGCCACTCAAATCTTTGGATTAG
Cagβ(S337A)-R-pet32a	CTAATCCAAAGATTTGAGTGGCAAAATCCCCTCCAC
Cagβ(T338A)-F-pet32a	GGAGGGGATTTTTCCGCTCAAATCTTTGGATTAG
Cagβ(T338A)-R-pet32a	CTAATCCAAAGATTTGAGCGGAAAAATCCCCTCC
Cagβ(E350A)-F-pet32a	GCTCGTGTTCCCTGCAAGACCTAATGAAAAAG
Cagβ(E350A)-R-pet32a	CTTTTTCATTAGGTCTTGCAGGGAACACGAGC
Cagβ(D356A)-F-pet32a	GAAAGACCTAATGAAAAAGCTCCTTTCTTTAGCAATCAAG
Cagβ(D356A)-R-pet32a	CTTGATTGCTAAAGAAAGGAGCTTTTTCATTAGGTCTTTC
Cagβ(F358A)-F-pet32a	CCTAATGAAAAAGATCCTGCCTTTAGCAATCAAGCG
Cagβ(F358A)-R-pet32a	CGCTTGATTGCTAAAGGCAGGATCTTTTTCATTAGG
Cagβ(N361A)-F-pet32a	GATCCTTTCTTTAGCGCTCAAGCGCGAAATCTTTTTG
Cagβ(N361A)-R-pet32a	CAAAAAGATTTCGCGCTTGAGCGCTAAAGAAAGGATC
Cagβ(N365A)-F-pet32a	GCAATCAAGCGCGAGCTCTTTTTGTCATCAATTGC
Cagβ(N365A)-R-pet32a	GCAATTGATGACAAAAAGAGCTCGCGCTTGATTGC
Cagβ(Y374F)-F-pet32a	CATCAATTGCAATATTTTCAGGGATCTCATGTGG

Cagβ(Y374F)-R-pet32a	CCACATGAGATCCCTGAAAATATTGCAATTGATG
Cagβ(R375A)-F-pet32a	CAATTGCAATATTTACGCGGATCTCATGTGGAC
Cagβ(R375A)-R-pet32a	GTCCACATGAGATCCGCGTAAATATTGCAATTG
Cagβ(D376A)-F-pet32a	GCAATATTTACAGGGCTCTCATGTGGACTAAAAAGG
Cagβ(D376A)-R-pet32a	CCTTTTTAGTCCACATGAGAGCCCTGTAAATATTGC
Cagβ(K388A)-F-pet32a	GGGCTTGAGTTTGTCGCAAGAAAAAAAATCATCATG
Cagβ(K388A)-R-pet32a	CATGATGATTTTTTTTCTTGCGACAAACTCAAGCCC
Cagβ(E396A)-F-pet32a	GAAAAAAAATCATCATGCCTGCAACACCCACGATGTTTTTC
Cagβ(E396A)-R-pet32a	GAAAAACATCGTGGGTGTTGCAGGCATGATGATTTTTTTTC
Cagβ(N449A)-F-pet32a	CTTAGTCCTGCCACTAGAGCCATGTGGAATAGCTTC
Cagβ(N449A)-R-pet32a	GAAGCTATTCCACATGGCTCTAGTGGCAGGACTAAG
Cagβ(N452A)-F-pet32a	CACTAGAAACATGTGGGCTAGCTTCAAGACAATGG
Cagβ(N452A)-R-pet32a	CCATTGTCTTGAAGCTAGCCCACATGTTTCTAGTG
Cagβ(S453A)-F-pet32a	CTAGAAACATGTGGAATGCCTTCAAGACAATGGGC
Cagβ(S453A)-R-pet32a	GCCCATTGTCTTGAAGGCATTCCACATGTTTCTAG
Cagβ(E462A)-F-pet32a	CAATGGGCGGCGCTAGAGCAACTTATAGCTCGGTTC
Cagβ(E462A)-R-pet32a	GAACCGAGCTATAAGTTGCTCTAGCGCCGCCCATTG
Cagβ-R241A-F-pet32a	TGGCTTGATTGCTCCTACTGCTAGCGGTAAGGGGGTGG
Cagβ-R241A-R-pet32a	CCACCCCCTTACCGCTAGCAGTAGGAGCAATCAAGCCA
Cagβ-R241K-F-pet32a	ATTGCTCCTACTAAAAGCGGTAAGGGGGTGGGT
Cagβ-R241K-R-pet32a	ACCCACCCCCTTACCGCTTTTAGTAGGAGCAAT
Cagβ-K244A-F-pet32a	GCTCCTACTAGAAGCGGTGCGGGGGTGGGTTTCATCAT
Cagβ-K244A-R-pet32a	ATGATGAAACCCACCCCCGCACCGCTTCTAGTAGGAGC
Cagβ-E551A-F-pet32a	GATGCTCATGGACGCATTCACTTTATGTGGCTA
Cagβ-E551A-R-pet32a	TAGCCACATAAAGTGAATGCGTCCATGAGCATC
Cagβ-E551Q-F-pet32a	GATGCTCATGGACCAATTCACTTTATGTGGCTA
Cagβ-E551Q-R-pet32a	TAGCCACATAAAGTGAATTGGTCCATGAGCATC
